# Rethinking schizophrenia: insights from genomics and implications for research

**DOI:** 10.1038/s41380-026-03654-9

**Published:** 2026-05-21

**Authors:** Michael J. Owen, Michael C. O’Donovan

**Affiliations:** 1 Centre for Neuropsychiatric Genetics and Genomics, Division of Psychological Medicine and Clinical Neurosciences, Cardiff University, Cardiff, UK; 2 Neuroscience & Mental Health Innovation Institute, Cardiff University, Cardiff, UK

**Keywords:** Schizophrenia, Genetics

## Abstract

Recent genomic research, considered in the wider context of knowledge from outside genomics, provides significant conceptual insights into the aetiology and pathogenesis of schizophrenia. The evidence indicates that genetic risk is expressed across the lifespan, from foetal development through to adulthood, and involves multiple neuronal types and brain regions. Schizophrenia appears to be primarily a neuronal disorder, with synaptic dysfunction playing a central role in pathogenesis both during development and in mature adult brain function, alongside earlier non-synaptic neurodevelopmental mechanisms. Importantly, non-familial genetic and environmental factors substantially influence neurodevelopmental impairment, and this is often reflected in cognitive performance falling below familial expectations. Cognitive deficits and structural brain abnormalities are weakly correlated with familial genetic risk and are better understood as markers of neurodevelopmental vulnerability rather than causal mediators. Genomic findings also position schizophrenia within a neurodevelopmental continuum, spanning childhood-onset disorders to adult-onset psychiatric conditions, and suggest heterogeneity within schizophrenia, with some cases exhibiting stronger neurodevelopmental involvement. These findings challenge notions that schizophrenia can be ascribed to, or understood by studying, dysfunction in particular neuronal types, brain regions or circuits, or to defects at a particular stage of neurodevelopment. While schizophrenia appears to be predominantly a neuronal disorder, pathophysiology appears to be manifest widely across time and space, and in different neuronal types across the adult and foetal brain. Moreover, despite schizophrenia’s high heritability, there is mounting evidence that non-familial genetic and environmental factors play important roles in the neurodevelopmental processes that impact on schizophrenia risk. Finally, variation in the impact of the neurodevelopmental factors appears to be key to understanding some of the heterogeneity within schizophrenia and the relationship between schizophrenia and other conditions. These observations have profound implications for future research, particularly in clarifying pathogenic mechanisms and refining diagnostic frameworks.

## Introduction

A plethora of genomic studies of schizophrenia has been published over the past 20 years. These are often highly technical and published in either general scientific or highly specialist journals. There have also been several recent reviews of this literature including our own [1–3]. These have tended to focus, appropriately, on summarising the many novel findings rather than considering the broader conceptual implications and relevance to many aspects of schizophrenia research.

In this article, which is aimed at readers who may be unfamiliar with genomics research, we build on previous reviews to briefly emphasise what we see as the most important findings and cover in more detail their conceptual implications for understanding schizophrenia. Genomics offers a particularly powerful approach to answering some fundamental questions about the condition because genetics is the strongest known risk factor and interpretation of genetic findings is not clouded by the possibility of reverse causation. We argue that genomic studies, when considered in the broader context of knowledge from outside genomics, reveal several novel insights that challenge existing notions about schizophrenia. These include where and when it is manifest in the brain, its relationship to other neurodevelopmental and psychiatric disorders, heterogeneity, and the role of non-familial genetic and environmental risk factors. Our intention is not to provide yet another exhaustive review of genomic findings, but rather to consider what these findings might be telling us about schizophrenia and what the implications might be for future research and treatment.

## Where is schizophrenia risk manifest?

Genomic studies have revealed a complex polygenic architecture with hundreds of common risk variants as well as a smaller number of rare risk variants such as copy number variants (CNVs) and rare coding variants (RCVs) [1]. This allows researchers to ask whether the pattern of expression of the genes implicated by these findings or the proteins they encode point to risk being manifest in specific tissues or brain regions. Studies of this kind have shown that genes with high relative expression in the brain are enriched for risk variants rather than those with high relative expression in any other tissue. These findings suggest that schizophrenia risk variants primarily exert their effects via brain-related mechanisms rather than through effects on other organs or tissues [4, 5].

However, the genetic findings take us further than this [1, 6]. First, they implicate CNS neurons, both inhibitory and excitatory, rather than glia [4, 7]. Secondly, they implicate genes with high relative expression in neurons in most regions of the human brain [4, 7], perhaps explaining the lack of regional specificity of structural imaging studies (see below). Thirdly, the brain regions most strongly implicated in schizophrenia substantially overlap with brain regions that show high relative expression for genes implicated in bipolar disorder, depression, neuroticism and IQ [6, 7]. Fourthly, they implicate genes encoding proteins, such as the NMDA receptor and associated proteins, that are involved in biological processes that are fundamental to neuronal function and in particular synaptic structure and function [4, 8–10]. Taken together, these findings point to schizophrenia reflecting disturbances in neuronal and particularly synaptic function that are distributed across many brain regions, circuits and cell types [1, 11]. In other words, rather than being the result of dysfunction in a limited set of brain regions or circuits, schizophrenia may be best understood as resulting from fundamental, generalised disturbances in the way neurons function, communicate and develop, some of which are likely to overlap with those that are centrally involved in other psychiatric disorders as well as personality and cognitive traits.

This conclusion makes perfect sense when we consider the clinical manifestations of schizophrenia. Schizophrenia is a broad, heterogeneous syndromic diagnosis made solely on clinical observations. The core of positive, negative and disorganised symptoms which, by convention, form the basis of current diagnostic processes are diverse and likely reflect disturbances in brain function across multiple regions and circuits [12]. Moreover, the core symptoms of schizophrenia are frequently accompanied by non-diagnostic features such as a broad range of a cognitive impairments [13], affective symptoms, movement disorders and non-canonical sensory disturbances [11]. Finally, many imaging studies have confirmed that brain structural abnormalities, including reductions in cortical surface area and thickness and in the size of subcortical structures, are present in schizophrenia but these are widespread as well as being subtle and heterogenous [14–16]. These considerations seem entirely in line with our conclusion that underlying schizophrenia are widespread disruptions of brain function.

In the adult brain, although the genetic signal points to widespread neuronal pathology, sub-populations of excitatory neurons in hippocampus, amygdala and in cerebral cortex as well as of inhibitory neurons in cortex, amygdala and striatum show the most prominent genetic evidence, again many overlapping with cell populations implicated in other psychiatric disorders as well as cognitive and personality traits [7]. It seems reasonable to assume that disturbances in subsets of neurons, brain regions or circuits underlie specific symptoms of schizophrenia as well as cognitive impairments or other features associated with the disorder. However, importantly, these will be variably accompanied by more widespread neuronal pathophysiology associated with other symptoms and features of the broader schizophrenia syndrome and this has important implications for drawing causal inferences that we will come to later.

Altered dopaminergic neurotransmission is generally thought to be central to schizophrenia pathophysiology, particularly positive psychotic symptoms (delusions, hallucinations), and, until recently, all effective antipsychotics were dopamine DRD2 receptor antagonists [17]. Although the locus containing *DRD2* has a common variant association with schizophrenia [4], there is little evidence for a convergence of genetic signal onto a wider set of genes linked to dopaminergic transmission [18]. How to interpret this is unclear, but one possibility is that altered dopamine functioning in schizophrenia represents a final common pathway to psychotic symptoms, occurring secondary to primary aetiological events discussed above.

## When is schizophrenia risk manifest? Support for the neurodevelopmental hypothesis

**Fig. 1:**
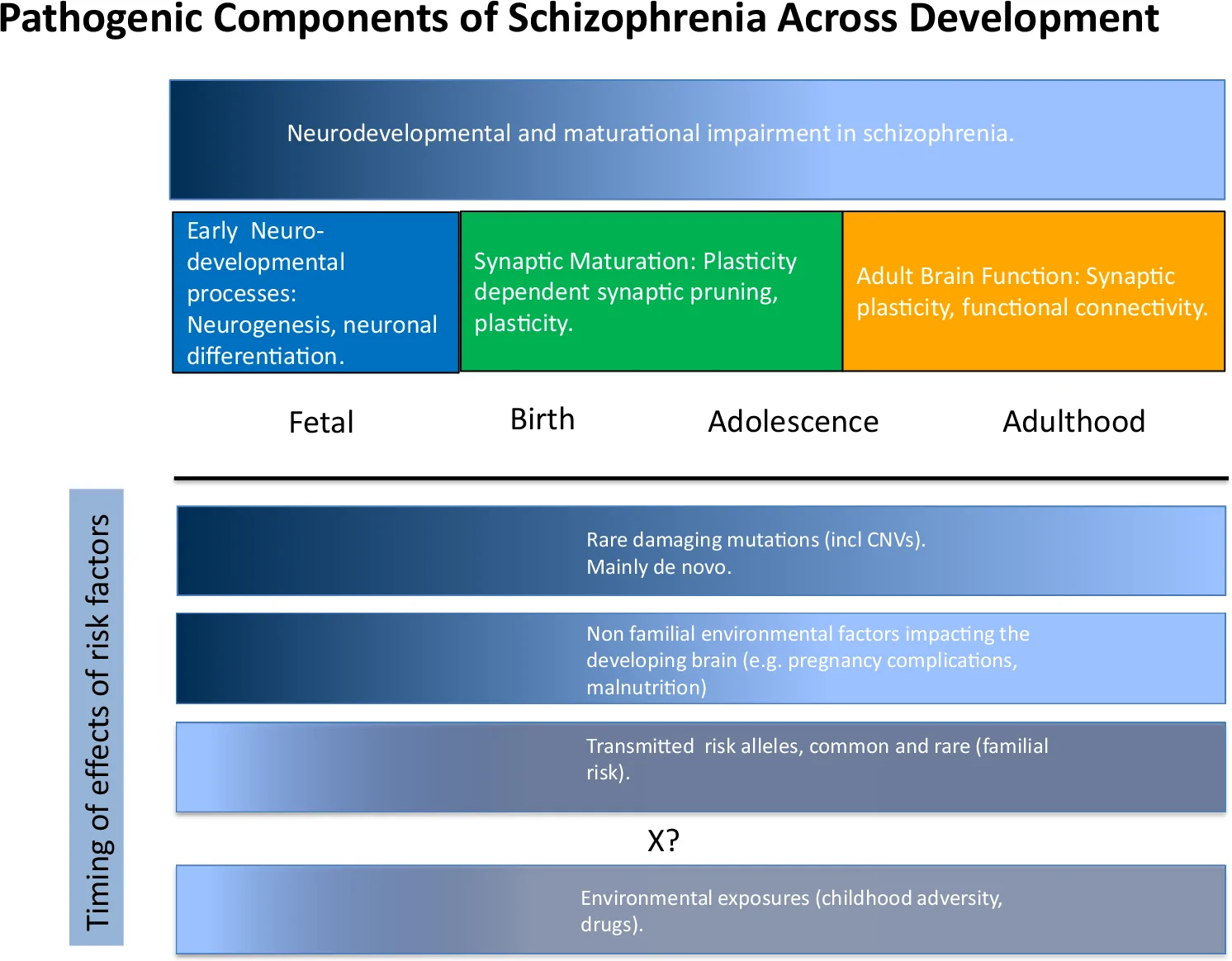
Pathogenic Components of Schizophrenia Across Development. A schematic representation of schizophrenia pathogenesis across the lifespan. The evidence reviewed points to three distinct components of pathogenesis impacting respectively on early neurodevelopment, synaptic maturation and adult function with abnormalities of synaptic function contributing to the latter two. There is a need now to understand how these components are mechanistically related and the interplay between them. Although disruption of synaptic mechanisms appears to be a common theme, there is evidence for distinct mechanisms early in neurodevelopment, such as neurogenesis and neuronal differentiation, before functioning synapses are formed (see main text for further discussion). The neurodevelopmental and maturational abnormalities are indexed by widespread but often subtle structural brain changes and premorbid cognitive function that is impaired relative to other family members. These do not appear to lie on the causal pathway to schizophrenia. The likely contribution of different classes of genetic and environmental risk is also shown. X?: Familial risk appears to interact with neurodevelopmental and maturational processes to increase risk of schizophrenia, but the nature of this interaction is unknown. Proximal environmental risk factors such as psychosocial adversity and drug abuse may impact on synaptic maturation if exposure occurs during adolescence as well as on adult brain function.

It has long been argued that schizophrenia is, at least in part, a neurodevelopmental disorder based on associations with premorbid developmental, cognitive and motor abnormalities and with environmental exposures in the prenatal period [19–21]. The so-called “neurodevelopmental hypothesis” has received much support from genomics. Genomic evidence for prenatal neurodevelopmental effects comes from findings that common alleles associated with schizophrenia are enriched for effects on gene expression in the human foetal brain [22]. Moreover, schizophrenia risk genes implicated by both common and rare genetic variants are enriched for those showing high relative expression in foetal neurons and include genes with known roles in neurodevelopment, particularly neurogenesis, neuronal differentiation and neurite outgrowth [4, 10, 23, 24]. Importantly, the common variant enrichments in genes with high relative expression in developing neurons are largely independent of those seen in neurons in adult brain [24], suggesting that the genetic signal in foetal neurons reflects a neurodevelopmental pathology that is distinct from the perturbations of neuronal function and particularly synaptic function that are inferred from studies of adult brain (Fig. 1).

Further support for neurodevelopmental mechanisms comes from the observation that the genes impacted by rare risk variants implicated in schizophrenia overlap those implicated in childhood neurodevelopmental disorders such as autism, ADHD and intellectual disability [25]. Moreover, not only do the genes implicated by rare risk alleles overlap, so too do specific rare risk alleles, including both CNVs and rare disruptive mutations [1]. Further, genes implicated by common allele associations are enriched for genes associated with rare disruptive mutations in childhood onset neurodevelopmental disorders [4, 10]. Together with the common allele genetic correlations between schizophrenia and neurodevelopmental disorders [6], these findings point to at least partial convergence of the effects of rare and common risk variants onto the same neurodevelopmental processes. As well as implicating neurodevelopmental processes in schizophrenia, these genetic overlaps with childhood neurodevelopmental disorders imply a degree of overlap in the primary molecular pathology. Finally, support for shared aetiology between schizophrenia and childhood neurodevelopmental disorders comes from evidence that many of the environmental risk factors for schizophrenia impact on the developing brain, and are shared with childhood neurodevelopmental disorders [26].

## Schizophrenia and other neurodevelopmental disorders: the neurodevelopmental continuum and gradient

The enrichment of rare risk mutations is not equal across neurodevelopmental disorders. It is strongest in intellectual disability (ID), then autism and ADHD, then schizophrenia and lower still in bipolar disorder [25]. These findings, and other evidence, led us to propose that schizophrenia is part of wider continuum of neurodevelopmental impairment with a gradient of decreasing impairment, indexed by cognitive impairment and age at manifestation, going from ID, through childhood neurodevelopmental conditions, schizophrenia, schizoaffective disorder and bipolar disorder [11, 25, 27, 28]. While the hypothesis of a continuum of neurodevelopmental disorders with a gradient of severity of neuropathology was proposed in the 1950s to describe the impact of obstetric complications and other factors associated with early cerebral insult [29], we expanded this to include the impact of rare genetic variants and to cross the nosological divide between childhood neurodevelopmental disorders and adult psychiatric conditions such as schizophrenia and bipolar disorder. As well as accumulating evidence from genomics [1, 6], the neurodevelopmental gradient model has received support from comparative studies of cognition and brain structure [30, 31]. Lastly, there is evidence that schizophrenia risk is better indexed by the degree to which premorbid cognitive and educational ability falls short of that expected based on the performance of family members rather than by cognitive ability per se [32, 33]. The magnitude of this effect is much greater in schizophrenia than bipolar disorder but less than that in autism [34]. This not only supports the neurodevelopmental gradient model but also suggests that non-familial factors, such as de novo mutations and environmental exposures, must play an important role in causing the neurodevelopmental impairments seen in these conditions, a consideration to which we will return.

## Relationship with other psychiatric disorders

The genetic findings discussed above, coming predominantly from rare risk variants, have positioned schizophrenia on a neurodevelopmental continuum between childhood neurodevelopmental conditions and bipolar disorder on a gradient of severity. However, schizophrenia also shows genetic correlations for common alleles with many other psychiatric disorders, particularly bipolar disorder and to a lesser extent depression, likely reflecting substantial pleiotropy [6, 35, 36]. The pleiotropic effects of risk alleles may explain why schizophrenia, bipolar disorder and depression share many transdiagnostic clinical features. Cross disorder genomic research is a burgeoning area with several new approaches being applied [6, 37], but initial findings suggest that groups of symptoms such as psychotic, manic or depressive symptoms are influenced by the same genetic variants regardless of the primary diagnoses, that each group of symptoms has partly independent underling genetic risk, and by implication, partly independent pathophysiology, and the aggregate of these helps determine the mix of symptoms seen in any individual case regardless of the primary diagnostic label [6].

Nevertheless, from the clinical and research perspectives, comparison of within and between diagnosis genetic correlations suggests that, while current diagnostic criteria may not define biologically distinct conditions, they do identify groups of cases whose members have on average more in common with each other than they do with groups with other psychiatric diagnoses [1].

## Genetic risk also manifests postnatally

Alleles conferring risk of schizophrenia are enriched among those that affect gene expression in the adult brain and, as discussed above, schizophrenia risk genes are enriched for those with high expression specificity in adult neurons across many brain regions, an enrichment that is substantially independent of analogous findings in foetal brain (Fig. 1) [4, 23, 24, 38–40]. Moreover, schizophrenia genes implicated by rare mutations do not, as a group, show strong pre- or post-natal bias in brain expression [10].

There is evidence that schizophrenia risk genes impact on synaptic maturation during childhood and adolescence via activity dependent pruning, and on adult brain function via effects on synaptic plasticity (Fig. 1) [4, 8–10, 23, 41, 42]. Abnormalities in synaptic structure and function in schizophrenia and their impact on connectivity have long been suspected and supported by neuropharmacological and neuropathological studies and by clinical neuroscience [23, 43, 44]. This led to the formulation of the dysconnection hypothesis, which proposes that schizophrenia is the result of a defect in functional (synaptic) connectivity resulting from an aberrant modulation of synaptic efficacy (plasticity) [44]. This hypothesis has received support from recent genomic findings, theoretical neuroscience and computational psychiatry as well as data from functional neuroimaging and provides a putative mechanism for how defects in adult synaptic physiology could underlie some of the features of schizophrenia [44].

While the findings support the notion that genetic risk for schizophrenia is expressed in the adult brain and results in abnormalities of synaptic plasticity, given the important role that synaptic mechanisms play in brain development both pre-natally and in childhood and adolescence, disruption of synaptic function may contribute to both the neurodevelopmental and adult manifestations of schizophrenia risk [23, 44]. However, as we have seen, the common variant enrichments in developing foetal neurons are largely independent of those seen in neurons in adult brain strongly suggesting the existence of early aetiological neurodevelopmental mechanisms that are distinct from those implicated in adults [24]. Moreover, there is evidence that isoform utilization, spatiotemporal expression, and subcellular localization of ion channels and synaptic proteins implicated in neurodevelopmental disorders in the developing brain are different from that in adult brain, leading to the reasonable be inference that the same risk genes may subserve distinct roles as the nervous system develops [45].

In conclusion, we believe the evidence points to three distinct components of pathogenesis impacting respectively on early neurodevelopment, synaptic maturation and adult function with abnormalities of synaptic function contributing to the latter two (Fig. 1).

## Cognitive impairment and structural brain abnormalities are markers of neurodevelopmental impairment and do not lie on the causal pathway

It is well recognised that schizophrenia is associated with impairments across many domains of cognitive function including IQ and that low premorbid IQ is associated with increased risk of schizophrenia [33, 46]. Evidence from genetic and population studies suggests that premorbid cognitive impairment is a marker for underlying neurodevelopmental risk factors rather than a manifestation of prodromal illness [33, 47, 48]. Variation in premorbid cognition in schizophrenia is not substantially the result of common allele genetic risk for schizophrenia [33]. Indeed, as we have mentioned above, schizophrenia risk is better indexed by the degree to which cognitive ability falls short of familial expectations rather than by cognitive ability per se [32, 33]. Moreover, premorbid cognitive impairment appears to be more strongly associated with non-familial factors than with inherited factors that jointly influence risk to schizophrenia and cognitive ability, and these non-familial factors sensitise the individuals to familial risk [48]. These findings suggest that premorbid impairments in cognition do not lie on the causal pathway from familial risk to schizophrenia but rather are a marker of neurodevelopmental abnormalities that are substantially non-familial, but which increase the risk of schizophrenia and sensitise the individual to the effects of familial risk [32, 33, 48].

The widespread but relatively subtle structural brain abnormalities seen in schizophrenia are associated with cognitive impairment [49] and, like cognitive impairment, appear to be in part manifest premorbidly and to be neurodevelopmental in origin [50]. If, as we have seen, cognitive impairment in schizophrenia is more strongly associated with non-familial than familial factors and only weakly associated with common variant genetic risk for schizophrenia, then this raises the question of whether the same may be true for structural brain abnormalities. Interestingly several systematic reviews have found little or no association between common variant genetic risk for schizophrenia and structural brain changes [16, 51, 52]. In the most recent and largest of these [16], which covered a wide range of structural as well as functional imaging measures, the only consistent structural association of schizophrenia polygenic risk score (PRS) was with reduced whole brain cortical thickness. However, this was not seen in the largest study which was based on a community sample rather than cases of schizophrenia [53], consistent with the conclusion that reduced cortical thickness results from reverse causation as it is associated with illness severity and duration, as well as medication use [14]. Moreover, overall, even where positive effect sizes were seen they were small ( < 2% of variance), and there was evidence of a negative association between effect size and sample size suggesting publication bias. The authors conclude that cumulative genetic risk, at least as indexed by PRS, is not a potent predictor of structural brain variability in any of the measures studied, and point to the possibility that other genetic or environmental factors might drive variability in brain measures commonly implicated in schizophrenia [16]. In this regard it is interesting that rare genetic risk factors (CNVs and RCVs), many of which will have occurred de novo and hence not be familial, are robustly associated with structural brain changes and cognitive impairment with much larger effect sizes than any observed for schizophrenia PRS despite the fact that the latter captures a higher fraction of liability to the disorder [31, 54].

In summary, structural brain changes and premorbid cognitive impairment in schizophrenia are at best weakly associated with polygenic risk for schizophrenia and the evidence points to stronger influences coming from non-familial factors that may include environmental exposures and de novo genetic mutations. Structural brain abnormalities and premorbid cognitive impairment are probably best regarded as markers indexing the neurodevelopmental processes conferring risk to schizophrenia rather than being causally implicated in pathogenesis.

## Summary and implications

Findings from genomics, cognition and structural imaging, together with consideration of the breadth, complexity and heterogeneity of the schizophrenia syndrome and associated features suggest that schizophrenia is accompanied by widespread disruptions of brain function. Recent genomic findings also suggest, in accord with other evidence, that schizophrenia is associated both with disruptions of neurodevelopment and with a partly distinct and widespread disruption of neuronal and particularly synaptic dysfunction in adulthood.

Abnormalities in synaptic function are likely to impact many aspects of adult brain function from microcircuits to communication between brain regions, and to impact many brain functions at higher levels of organisation. This is in turn can be expected to result in defects in many observable aspects of mature brain function, including those that underlie the core diagnostic features of schizophrenia [44]. However these abnormalities in synaptic function, and their downstream consequences, appear to be superimposed on a separate neurodevelopmental component, indexed by cognitive impairment and structural brain abnormalities, that is not strongly familial. This neurodevelopmental component interacts with familial risk of schizophrenia so that those with high familial risk and evidence of a neurodevelopmental impairment have a higher risk of illness than those with a similarly high familial risk who do not have evidence of a neurodevelopmental impairment [32]. The neurodevelopmental impairments may arise at least in part from the roles many synaptic genes play in brain development, but there is also evidence that separate mechanisms may be at play including those involved with neurogenesis and neuronal differentiation. Finally, it appears that neurodevelopmental factors play a more important role in some cases of schizophrenia than in others as indexed by the severity of cognitive impairment, the extent of structural brain abnormalities, as well as earlier age at onset and premorbid features [55]. In other words, there is a continuum of neurodevelopmental impairment across schizophrenia. Moreover, there is evidence, discussed above, that this is part of wider continuum with a gradient of decreasing neurodevelopmental impairment going from ID, through childhood neurodevelopmental conditions, schizophrenia, schizoaffective disorder and bipolar disorder.

These conclusions have several important implications for research, that we will now briefly consider. First, to understand the pathogenesis of schizophrenia, researchers will need to be aware that there may be different pathogenic mechanisms at play across development with evidence for distinct processes occurring early in neurodevelopment, during synaptic maturation and in the mature adult brain. It will be important for future functional genomic studies to consider this possibility when seeking to gain biological insights from genomic data. There are opportunities from advances in cell and organoid models combined with genome engineering to understand the nature of neuronal and synaptic dysfunctions at a molecular and circuit level [56], although these are currently more suitable for studying early developmental stages. There are also opportunities using functional imaging approaches in both animal models and human studies to understand how alterations in synaptic function impact on functional brain connectivity and how these alterations relate to particular symptoms and other features of the schizophrenia syndrome, but again we argue that developmental considerations will be important.

Secondly, while individual symptoms and other features may be associated with dysfunction in specific brain regions and circuits, these will be present against a background of more widely distributed neuronal pathology associated with other symptoms and outcomes associated with schizophrenia. This means that there are important, but still poorly appreciated, implications for research aiming to identify neurobiological mechanisms mediating the effects of genetic and other risk factors on behavioural or symptomatic outcomes [35]. Put simply this means that just because a neurobiological abnormality in a particular brain region or circuit is present in people with schizophrenia, or in a model system, this does not allow one to assume that this mediates a particular outcome such as psychosis or cognitive impairment [35]. This issue has been discussed more fully elsewhere in relation to both human endophenotypes and model systems [35, 57, 58].

Thirdly, researchers should be cautious in assuming that the effects of risk genes are mediated through cognitive impairment or structural brain abnormalities. Specifically, it seems that common allele genetic risk, which is the strongest determinant of familial risk, is not strongly associated with either outcome. Rather the evidence points to a role for non-familial factors and suggests that cognitive impairment and structural brain abnormalities may be markers of underlying neurodevelopmental impairment and do not lie on the causal pathway between familial risk and the core features of schizophrenia.

Fourthly, there is an imperative to understand the non-familial factors that underlie neurodevelopmental impairment. These are likely to include environmental exposures that impact the developing brain [26]. There is also strong evidence these non-familial factors include de novo SNV or CNV mutations, but to date these have been implicated in fewer than 5% of cases [1]. However, non-familial genetic factors may include as yet unidentified de novo mutations including classes of mutation that have not been systematically assayed in schizophrenia such as rare structural variants (other than CNVs), non-coding mutations, somatic mutations [59] and rare mutations that are incompletely penetrant. Studies of individuals, from both patient and population samples, who show evidence of cognitive ability that deviates negatively from familial expectations may be optimal for identifying these non-familial genetic risk factors and for studying their consequences.

Fifthly, the evidence suggests that the pathogenesis of schizophrenia reflects process operating across early neurodevelopment, synaptic maturation and adult function. There is a need now to understand how these components are mechanistically related and the interplay between them. Although disruption of synaptic mechanisms appears to be a common theme, there is evidence for distinct mechanisms early in neurodevelopment, such as neuronal differentiation [24, 60], before functioning synapses are formed. In addition, there is evidence that genes implicated in neurodevelopmental disorders, including schizophrenia, may have different functions early in neurodevelopment [45, 60]. Environmental risk factors also appear to act at different points of development with some, such as maternal infections, nutritional deficiencies, and pregnancy and birth complications, appearing to impact early on the developing brain and others, such as drug abuse and chronic social adversity, later in life [26, 61]. Again, the relationship between risk factors operating at different developmental stages needs to be understood as does the possible relationship with genetic risk including both genetic confounding and gene environment interactions [26].

It should also be noted that the balance between early neurodevelopmental, synaptic maturation and adult risk mechanisms may vary across and between psychotic disorders as we move across the neurodevelopmental gradient and explain some of the clinical heterogeneity, for example in cognitive impairment, among cases diagnosed with schizophrenia [23, 25]. This implies that research on endophenotypes, biomarkers and other biological correlates will be sensitive to case ascertainment and the extent to which it favours the inclusion of cases with a stronger neurodevelopmental component. It has also been proposed that the evidence for both pre- and post-natal contributions to the pathogenesis of schizophrenia suggests a two-pronged approach to intervention [23]; Prevention could focus upon maximising synaptic integrity during brain development through strategies to optimise nutrition and maternal care during pregnancy, with later treatments aiming to improve impairments in synaptic function after the onset of symptoms [23].

Finally, the evidence for overlapping genetic effects between schizophrenia and other neurodevelopmental and psychiatric disorders remind us of the limitations of current diagnostic approaches for defining biologically distinct conditions [6]. This points to the need for more research across diagnostic categories focussing on shared transdiagnostic features. This is beginning to reveal partly separate genetic risk for the major symptom categories, but there is a need now to extend this work to other risk factors and to integrate greater clinical granularity with neuroscience measures and other potential biomarkers. Work of this nature in has identified evidence for different biotypes that cut across current diagnoses of psychotic disorders and further work of this kind incorporating genetic measures is likely to be fruitful [62]. Moreover, our discussion above suggests that work attempting to define more biologically valid diagnostic groupings will need to take a developmental perspective and that markers of neurodevelopmental involvement such as cognitive impairment, age at onset and premorbid impairments may turn out to be important stratifiers. There is also evidence that geneticists need to pay greater attention to phenotype definition since more superficial phenotyping, for example from questionnaires or medical records, leads to less circumscribed genetic signals than phenotyping based on clinical interviews by a trained rater [6, 37]. The inclusion of less stringently defined cases will confound efforts to investigate heterogeneity and transdiagnostic relationships, and this has led to calls for new cohorts for genetic research in psychiatry to be established with deeper and more diverse phenotypes [6, 37].

## Conclusions

The findings we have reviewed challenge notions that schizophrenia can be ascribed solely to dysfunction in particular neuronal types, brain regions or circuits, or to defects at a particular stage of neurodevelopment. While schizophrenia appears to be predominantly a neuronal disorder, pathophysiology appears to be manifest widely across time and space, from foetal life through to adulthood and in different neuronal types across the adult and foetal brain. Moreover, despite schizophrenia’s high heritability, there is mounting evidence that non-familial genetic and environmental factors play important roles in the neurodevelopmental processes that impact on schizophrenia risk. Finally, variation in the impact of the neurodevelopmental factors appears to be key to understanding some of the heterogeneity within schizophrenia and the relationship between schizophrenia and other conditions.

## Data Availability

No new data were generated for this article

## References

[1] Owen MJ, Legge SE, Rees E, Walters JTR, O’Donovan MC. Genomic findings in schizophrenia and their implications. Mol Psychiatry. 2023;28:3638–47. 37853064 10.1038/s41380-023-02293-8 PMC10730422

[2] Andreassen OA, Hindley GFL, Frei O, Smeland OB. New insights from the last decade of research in psychiatric genetics: discoveries, challenges and clinical implications. World Psychiatry. 2023;22:4–24. 36640404 10.1002/wps.21034 PMC9840515

[3] Sullivan PF, Yao S, Hjerling-Leffler J. Schizophrenia genomics: genetic complexity and functional insights. Nat Rev Neurosci. 2024;25:611–24. 39030273 10.1038/s41583-024-00837-7

[4] Trubetskoy V, Pardiñas AF, Qi T, Panagiotaropoulou G, Awasthi S, Bigdeli TB, et al. Mapping genomic loci implicates genes and synaptic biology in schizophrenia. Nature. 2022;604:502–8. 35396580 10.1038/s41586-022-04434-5 PMC9392466

[5] Kendler KS. Are psychiatric disorders brain diseases?-a new look at an old question. JAMA Psychiatry. 2024;81:325–6. 38416478 10.1001/jamapsychiatry.2024.0036

[6] Owen MJ, Bray NJ, Walters JTR, O’Donovan MC. Genomics of schizophrenia, bipolar disorder and major depressive disorder. Nat Rev Genet. 2025. 10.1038/s41576-025-00843-0 40355602

[7] Yao S, Harder A, Darki F, Chang YW, Li A, Nikouei K, et al. Connecting genomic results for psychiatric disorders to human brain cell types and regions reveals convergence with functional connectivity. Nat Commun. 2025;16:395. 39755698 10.1038/s41467-024-55611-1 PMC11700164

[8] Kirov G, Pocklington AJ, Holmans P, Ivanov D, Ikeda M, Ruderfer D, et al. De novo CNV analysis implicates specific abnormalities of postsynaptic signalling complexes in the pathogenesis of schizophrenia. Mol Psychiatry. 2012;17:142–53. 22083728 10.1038/mp.2011.154 PMC3603134

[9] Hall J, Trent S, Thomas KL, O’Donovan MC, Owen MJ. Genetic risk for schizophrenia: convergence on synaptic pathways involved in plasticity. Biol Psychiatry. 2015;77:52–8. 25152434 10.1016/j.biopsych.2014.07.011

[10] Singh T, Poterba T, Curtis D, Akil H, Al Eissa M, Barchas JD, et al. Rare coding variants in ten genes confer substantial risk for schizophrenia. Nature. 2022;604:509–16. 35396579 10.1038/s41586-022-04556-w PMC9805802

[11] Owen MJ, Legge SE. The nature of schizophrenia: As broad as it is long. Schizophr Res. 2022;242:109–12. 34756599 10.1016/j.schres.2021.10.012

[12] Tandon R, Gaebel W, Barch DM, Bustillo J, Gur RE, Heckers S, et al. Definition and description of schizophrenia in the DSM-5. Schizophr Res. 2013;150:3–10. 23800613 10.1016/j.schres.2013.05.028

[13] Green MF, Horan WP, Lee J. Nonsocial and social cognition in schizophrenia: current evidence and future directions. World Psychiatry. 2019;18:146–61. 31059632 10.1002/wps.20624 PMC6502429

[14] van Erp TGM, Walton E, Hibar DP, Schmaal L, Jiang W, Glahn DC, et al. Cortical brain abnormalities in 4474 individuals with schizophrenia and 5098 control subjects via the Enhancing Neuro Imaging Genetics through Meta Analysis (ENIGMA) Consortium. Biol Psychiatry. 2018;84:644–54. 29960671 10.1016/j.biopsych.2018.04.023 PMC6177304

[15] van Erp TG, Hibar DP, Rasmussen JM, Glahn DC, Pearlson GD, Andreassen OA, et al. Subcortical brain volume abnormalities in 2028 individuals with schizophrenia and 2540 healthy controls via the ENIGMA consortium. Mol Psychiatry. 2016;21:585. 26283641 10.1038/mp.2015.118 PMC5751698

[16] Jameei H, Rakesh D, Zalesky A, Cairns MJ, Reay WR, Wray NR, et al. Linking polygenic risk of schizophrenia to variation in magnetic resonance imaging brain measures: a comprehensive systematic review. Schizophr Bull. 2024;50:32–46. 37354489 10.1093/schbul/sbad087 PMC10754175

[17] Murray RM, Alameda L, Dima A, Oliver D Beyond Kraepelin: An Aetiological Approach to the Typology and Treatment of Psychosis. In: Ikkos G, Becker T (eds). *Psychiatry after Kraepelin: Ambition Images Practices 1926-2026*. Cham: Springer Nature Switzerland, 2026, pp 417-48.

[18] Frei O, Hindley G, Shadrin AA, van der Meer D, Akdeniz BC, Hagen E, et al. Improved functional mapping of complex trait heritability with GSA-MiXeR implicates biologically specific gene sets. Nat Genet. 2024;56:1310–8. 38831010 10.1038/s41588-024-01771-1 PMC11759099

[19] Weinberger DR The pathogenesis of schizophrenia: a neurodevelopmental theory. In: Nasrallah HA, Weinberger DR (eds). *The Neurology of schizophrenia*. Amsterdam: Elsevier, 1986, pp 387-405.

[20] Weinberger DR. Implications of normal brain development for the pathogenesis of schizophrenia. Arch Gen Psychiatry. 1987;44:660–9. 3606332 10.1001/archpsyc.1987.01800190080012

[21] Murray RM, Lewis SW. Is schizophrenia a neurodevelopmental disorder? Br Med J (Clin Res Ed). 1987;295:681–2. 3117295 10.1136/bmj.295.6600.681 PMC1247717

[22] O’Brien HE, Hannon E, Hill MJ, Toste CC, Robertson MJ, Morgan JE, et al. Expression quantitative trait loci in the developing human brain and their enrichment in neuropsychiatric disorders. Genome Biol. 2018;19:194. 30419947 10.1186/s13059-018-1567-1 PMC6231252

[23] Hall J, Bray NJ. Schizophrenia genomics: convergence on synaptic development, adult synaptic plasticity, or both? Biol Psychiatry. 2022;91:709–17. 34974922 10.1016/j.biopsych.2021.10.018 PMC8929434

[24] Cameron D, Mi D, Vinh NN, Webber C, Li M, Marín O, et al. Single-nuclei RNA sequencing of 5 regions of the human prenatal brain implicates developing neuron populations in genetic risk for schizophrenia. Biol Psychiatry. 2023;93:157–66. 36150908 10.1016/j.biopsych.2022.06.033 PMC10804933

[25] Owen MJ, O’Donovan MC. Schizophrenia and the neurodevelopmental continuum:evidence from genomics. World Psychiatry. 2017;16:227–35. 28941101 10.1002/wps.20440 PMC5608820

[26] Owen MJ, Sawa A, Mortensen PB. Schizophrenia. Lancet. 2016;388:86–97. 26777917 10.1016/S0140-6736(15)01121-6 PMC4940219

[27] Craddock N, Owen MJ. The Kraepelinian dichotomy - going, going… but still not gone. Br J Psychiatry. 2010;196:92–95. 20118450 10.1192/bjp.bp.109.073429 PMC2815936

[28] Owen MJ. New approaches to psychiatric diagnostic classification. Neuron. 2014;84:564–71. 25442935 10.1016/j.neuron.2014.10.028

[29] Lilienfeld AM, Pasamanick B, Rogers M. Relationship between pregnancy experience and the development of certain neuropsychiatric disorders in childhood. Int J Epidemiol. 2016;45:311–6. 27174830 10.1093/ije/dyw067

[30] Lynham AJ, Hubbard L, Tansey KE, Hamshere ML, Legge SE, Owen MJ, et al. Examining cognition across the bipolar/schizophrenia diagnostic spectrum. J Psychiatry Neurosci. 2018;43:245–53. 29947606 10.1503/jpn.170076 PMC6019354

[31] Cheon EJ, Bearden CE, Sun D, Ching CRK, Andreassen OA, Schmaal L, et al. Cross disorder comparisons of brain structure in schizophrenia, bipolar disorder, major depressive disorder, and 22q11.2 deletion syndrome: A review of ENIGMA findings. Psychiatry Clin Neurosci. 2022;76:140–61. 35119167 10.1111/pcn.13337 PMC9098675

[32] Kendler KS, Ohlsson H, Mezuk B, Sundquist JO, Sundquist K. Observed cognitive performance and deviation from familial cognitive aptitude at age 16 years and ages 18 to 20 years and risk for schizophrenia and bipolar illness in a Swedish national sample. JAMA Psychiatry. 2016;73:465–71. 27028264 10.1001/jamapsychiatry.2016.0053

[33] Owen MJ, O’Donovan MC. The genetics of cognition in schizophrenia. Genomic Psychiatry. 2024;1:28–35.

[34] Kendler KS, Ohlsson H, Keefe RSE, Sundquist K, Sundquist J. The joint impact of cognitive performance in adolescence and familial cognitive aptitude on risk for major psychiatric disorders: a delineation of four potential pathways to illness. Mol Psychiatry. 2018;23:1076–83. 28416810 10.1038/mp.2017.78 PMC5647225

[35] O’Donovan MC, Owen MJ. The implications of the shared genetics of psychiatric disorders. Nat Med. 2016;22:1214–9. 27783064 10.1038/nm.4196

[36] Consortium B, Anttila V, Bulik-Sullivan B, Finucane HK, Walters RK, Bras J, et al. Analysis of shared heritability in common disorders of the brain. Science. 2018;360:eaap8757. 29930110 10.1126/science.aap8757 PMC6097237

[37] Grotzinger AD, Mallard TT, Akingbuwa WA, Ip HF, Adams MJ, Lewis CM, et al. Genetic architecture of 11 major psychiatric disorders at biobehavioral, functional genomic and molecular genetic levels of analysis. Nat Genet. 2022;54:548–59. 35513722 10.1038/s41588-022-01057-4 PMC9117465

[38] Richards AL, Jones L, Moskvina V, Kirov G, Gejman PV, Levinson DF, et al. Schizophrenia susceptibility alleles are enriched for alleles that affect gene expression in adult human brain. Mol Psychiatry. 2012;17:193–201. 21339752 10.1038/mp.2011.11 PMC4761872

[39] Wang D, Liu S, Warrell J, Won H, Shi X, Navarro FCP, et al. Comprehensive functional genomic resource and integrative model for the human brain. Science. 2018;362:eaat8464. 30545857 10.1126/science.aat8464 PMC6413328

[40] Bryois J, Calini D, Macnair W, Foo L, Urich E, Ortmann W, et al. Cell-type-specific cis-eQTLs in eight human brain cell types identify novel risk genes for psychiatric and neurological disorders. Nat Neurosci. 2022;25:1104–12. 35915177 10.1038/s41593-022-01128-z

[41] Pocklington AJ, Rees E, Walters JT, Han J, Kavanagh DH, Chambert KD, et al. Novel findings from CNVs implicate inhibitory and excitatory signaling complexes in schizophrenia. Neuron. 2015;86:1203–14. 26050040 10.1016/j.neuron.2015.04.022 PMC4460187

[42] Howes OD, Onwordi EC. The synaptic hypothesis of schizophrenia version III: a master mechanism. Mol Psychiatry. 2023;28:1843–56. 37041418 10.1038/s41380-023-02043-w PMC10575788

[43] Owen MJ, O’Donovan MC, Harrison PJ. Schizophrenia: a genetic disorder of the synapse? Bmj. 2005;330:158–9. 15661762 10.1136/bmj.330.7484.158 PMC544978

[44] Friston K, Brown HR, Siemerkus J, Stephan KE. The dysconnection hypothesis (2016). Schizophr Res. 2016;176:83–94. 27450778 10.1016/j.schres.2016.07.014 PMC5147460

[45] Panagiotakos G, Pasca SP. A matter of space and time: Emerging roles of disease-associated proteins in neural development. Neuron. 2022;110:195–208. 34847355 10.1016/j.neuron.2021.10.035 PMC8776599

[46] McCutcheon RA, Keefe RSE, McGuire PK. Cognitive impairment in schizophrenia: aetiology, pathophysiology, and treatment. Mol Psychiatry. 2023;28:1902–18. 36690793 10.1038/s41380-023-01949-9 PMC10575791

[47] Khandaker GM, Barnett JH, White IR, Jones PB. A quantitative meta-analysis of population-based studies of premorbid intelligence and schizophrenia. Schizophr Res. 2011;132:220–7. 21764562 10.1016/j.schres.2011.06.017 PMC3485562

[48] Kendler KS, Ohlsson H, Sundquist J, Sundquist K. IQ and schizophrenia in a Swedish national sample: their causal relationship and the interaction of IQ with genetic risk. Am J Psychiatry. 2015;172:259–65. 25727538 10.1176/appi.ajp.2014.14040516 PMC4391822

[49] Khalil M, Hollander P, Raucher-Chéné D, Lepage M, Lavigne KM. Structural brain correlates of cognitive function in schizophrenia: A meta-analysis. Neurosci Biobehav Rev. 2022;132:37–49. 34822878 10.1016/j.neubiorev.2021.11.034

[50] Karlsgodt KH, Sun D, Cannon TD. Structural and functional brain abnormalities in schizophrenia. Curr Dir Psychol Sci. 2010;19:226–31. 25414548 10.1177/0963721410377601 PMC4235761

[51] van der Merwe C, Passchier R, Mufford M, Ramesar R, Dalvie S, Stein DJ. Polygenic risk for schizophrenia and associated brain structural changes: A systematic review. Compr Psychiatry. 2019;88:77–82. 30529765 10.1016/j.comppsych.2018.11.014

[52] Cattarinussi G, Delvecchio G, Sambataro F, Brambilla P. The effect of polygenic risk scores for major depressive disorder, bipolar disorder and schizophrenia on morphological brain measures: a systematic review of the evidence. J Affect Disord. 2022;310:213–22. 35533776 10.1016/j.jad.2022.05.007

[53] Stauffer EM, Bethlehem RAI, Warrier V, Murray GK, Romero-Garcia R, Seidlitz J, et al. Grey and white matter microstructure is associated with polygenic risk for schizophrenia. Mol Psychiatry. 2021;26:7709–18. 34462574 10.1038/s41380-021-01260-5 PMC8872982

[54] Hubbard L, Rees E, Morris DW, Lynham AJ, Richards AL, Pardiñas AF, et al. Rare copy number variants are associated with poorer cognition in schizophrenia. Biol Psychiatry. 2021;90:28–34. 33678419 10.1016/j.biopsych.2020.11.025

[55] Rapoport JL, Addington AM, Frangou S, Psych MR. The neurodevelopmental model of schizophrenia: update 2005. Mol Psychiatry. 2005;10:434–49. 15700048 10.1038/sj.mp.4001642

[56] Brennand KJ. Using stem cell models to explore the genetics underlying psychiatric disorders: linking risk variants, genes, and biology in brain disease. Am J Psychiatry. 2022;179:322–8. 35491564 10.1176/appi.ajp.20220235

[57] Walters JT, Owen MJ. Endophenotypes in psychiatric genetics. Mol Psychiatry. 2007;12:886–90. 17895920 10.1038/sj.mp.4002068

[58] Kendler KS, Neale MC. Endophenotype: a conceptual analysis. Mol Psychiatry. 2010;15:789–97. 20142819 10.1038/mp.2010.8 PMC2909487

[59] Maury EA, Jones A, Seplyarskiy V, Nguyen TTL, Rosenbluh C, Bae T, et al. Somatic mosaicism in schizophrenia brains reveals prenatal mutational processes. Science. 2024;386:217–24. 39388546 10.1126/science.adq1456 PMC11490355

[60] Sanders B, D’Andrea D, Collins MO, Rees E, Steward TGJ, Zhu Y, et al. Transcriptional programs regulating neuronal differentiation are disrupted in DLG2 knockout human embryonic stem cells and enriched for schizophrenia and related disorders risk variants. Nat Commun. 2022;13:27. 35031607 10.1038/s41467-021-27601-0 PMC8760302

[61] Stilo SA, Murray RM. Non-genetic factors in schizophrenia. Curr Psychiatry Rep. 2019;21:100. 31522306 10.1007/s11920-019-1091-3 PMC6745031

[62] Clementz BA, Assaf M, Sweeney JA, Gershon ES, Keedy SK, Hill SK, et al. Categorical and dimensional approaches for psychiatric classification and treatment targeting: considerations from psychosis biotypes. Adv Neurobiol. 2024;40:685–723. 39562461 10.1007/978-3-031-69491-2_23 PMC12054367

